# Successful single-center experiences of laser crossectomy in zone zero in Endovenous Laser Ablation (EVLA) for chronic venous insufficiency - a case series

**DOI:** 10.12688/f1000research.170645.1

**Published:** 2026-01-21

**Authors:** Taofan Taofan, Suko Ardiarto, Suci Indriani, Ruth Grace Aurora, Adelin Dhivi, Aris Munandar, Vekky Sariowan, Erwin Mulia, Fadhil Pratama, Rido Jati Kuncara, Yuyun Pamungkas, Kelvin Kohar, Matthew Sebastian, Aditya Rachman, Eric Ferdinand, Junichi Utoh, Marc Vuylsteke, Thomas Weiler, Iwan Dakota, Bambang Widyantoro

**Affiliations:** 1 Department of Vascular Medicine, National Cardiovascular Center Harapan Kita Hospital, Jakarta, Indonesia; 2 Cardiologist Fellow/Resident in Vascular Medicine, Department of Vascular Medicine, National Cardiovascular Center Harapan Kita Hospital, Jakarta, Indonesia, Jakarta, Indonesia; 3 National Cardiovascular Center Harapan Kita Hospital, Jakarta, Indonesia; 4 Assistant Researcher, Department of Cardiology and Vascular Medicine, Department of Vascular Medicine, National Cardiovascular Center Harapan Kita Hospital, Jakarta, Indonesia, Jakarta, Indonesia; 5 Department of Vascular Surgery, Kumamoto Kekkan Vascular Clinic, Kumamoto, Japan; 6 Department of Vascular Surgery, Sint-Andriesziekenhuis Tielt, Tielt, Belgium; 7 Department of Vascular Surgery, Venenzentrum Pforzheim, Pforzheim, 75179, Germany

**Keywords:** Endovenous laser ablation, laser crossectomy, chronic venous insufficiency, varicose vein, safety, efficacy Main body

## Abstract

**Background:**

Chronic venous insufficiency (CVI) is a prevalent and underdiagnosed disease affecting over 25 million adults worldwide. Endovenous laser ablation (EVLA) remains the gold standard for great saphenous vein (GSV) insufficiency. However, conventional EVLA performed 1–2 cm distal to the saphenofemoral junction (SFJ) often leaves residual tributaries, increasing the risk of recurrence. Zone-zero laser crossectomy, defined as laser crossectomy performed exactly at the 0 cm zone of the SFJ (zone-zero ablation), had been proposed as a minimally invasive alternative technique to eliminate residual stumps and reduce varicose vein recurrence. This case series of zone-zero laser crossectomy aims to share our experiences and outcomes amongst 57 patients in our center.

**Methods:**

A total of 57 consecutive patients with symptomatic GSV insufficiency (CEAP C2–C6) underwent EVLA using a 1470 nm radial double-ring swift laser with zone-zero ablation at the SFJ. Procedural parameters, complications, and postoperative outcomes were recorded. Patients were followed at 7 days, 4, 8, and 12 weeks with physical and duplex ultrasound examinations.

**Results:**

The mean age was 61.6 years. No recanalization was observed during the 3-month follow-up, with duplex ultrasound confirming complete obliteration of treated GSVs. Postoperative complications included leg pain in 3 cases (5.26%), EHIT grade I–II in 1 case (1.75%), hyperpigmentation in 1 case (1.75%), and paresthesia in 1 case (1.75%). All complications were mild and managed conservatively.

**Conclusions:**

Zone-zero laser crossectomy is a safe and effective modification of EVLA for GSV insufficiency, demonstrating high obliteration rates and low complication rates. Larger cohorts with longer follow-up are needed to confirm durability.

## Introduction

Chronic venous insufficiency (CVI), a part of chronic venous disease, is defined as a chronic condition due to blockage of a vein, or blood leaking around the valves (reflux) of the veins, which results in venous hypertension. This disease is commonly underdiagnosed and progressively reduces patients’ quality of life. ^1^ To date, CVI affects more than 25 million adults, with various clinical manifestations, ranging from varicose vein to ulceration. ^2^

According to the European Society for Vascular Surgery (ESVS), endovenous thermal ablation (especially endovenous laser ablation) is still the gold standard in great saphenous vein insufficiency compared to other treatments. ^1^ In conventional technique of endovenous laser ablation, the operator performed vein ablation 1 to 2 cm away from saphenous femoral junction (SFJ) to minimize the risk of thrombosis progression. However, these residual tributary veins (stump), such as the superficial circumflex iliac vein, epigastric vein, and anterior accessory saphenous vein, are attributed to higher likelihood of recurrence and neovascularization. ^3^

A study by Nagib, et al introduced the “laser crossectomy” term, which is defined as ablation at 0 cm or exactly at SFJ. Their hypothesis is that by performing laser crossectomy in an accurate position, the operator will be able to eliminate any stumps, which correlates with less recurrence and complication rate. ^3^ The concept behind laser crossectomy is similar to the high ligation and stripping of GSV, but in a minimally invasive way. This technique also has been proven safe and effective in many studies, but comes with side effects, such as hematoma, cicatrix, and other aesthetic problems. ^4, 5^

This case series aims to evaluate the safety and efficacy of the novel technique “laser crossectomy” in a single center of National Cardiovascular Center Harapan Kita.

## Methods

### Case series

This article is a case series conducted in National Cardiovascular Center Harapan Kita, Indonesia, between February 2025 and June 2025. All data were collected and reviewed from medical records of patients who underwent EVLA of great saphenous vein incompetency using laser crossectomy technique. The patients were informed of the risks and benefits of the procedure and signed a written informed consent before undergoing said procedure.

### Preprocedural evaluation

Patients with symptomatic GSV insufficiency were evaluated in the hospital outpatient clinic. During the baseline evaluation, the following parameters were documented for each patient: age, sex, body mass index, symptoms and complications, family history, comorbidities, and physical examination findings. Lower extremity duplex ultrasound was also performed as a supporting examination. The presence of GSV insufficiency was diagnosed according to the reflux time, with >500 ms (0.5 s) findings considered significant reflux. Patients with significant reflux and CEAP clinical class C2-C6 were assigned for endovenous laser ablation therapy. Patients were consulted to the anesthesiologist and written informed consent was obtained.

### EVLA procedure

Before the procedure, patients were anesthetized using spinal anesthesia or Total Intravenous Anesthesia (TIVA) and placed in the supine position. All procedures were performed using 1470 nm radial double-ring swift diode laser fibers (ELVeS Biolitec, Germany-Austria). An intravenous delivery sheath (5-6 Fr) was inserted into the great saphenous vein (GSV), and the fiber tip was positioned precisely at the saphenous-femoral junction (0-Point Distance) with ultrasound guidance. A tumescent local anesthetic solution (consisting of 500 mg lidocaine, 1 mg of epinephrine, and 12.5 mEq sodium bicarbonate added to 1 L of 0.9% normal saline) was administered along the entire target vein using 23-G needle under ultrasound guidance with a tumescent pump. Using 1470nm EVLT with signal mode, the SFJ, a high dose of tumescent was given in order to ensure effective compression of the GSV around the catheter. Additionally, a high dose of energy (150 J) was given through the first segment of SFJ. The ablation was performed throughout the GSV by using a constant pullback speed of 0.14 cm/s (1 cm every 7 seconds) with Linear Endovenous Energy Density (LEED) of 40-60 J/cm under the guidance of ultrasound visualization of the bubble steam. The laser power, LEED, and laser energy were set according to the patients’ vein diameter and reflux severity. The total ablation length, treatment duration, total energy delivered, and applied tumescent during the laser procedure were documented. After the intervention, eccentric compression was applied using sterile gauze throughout the treated vein along with compression stockings. Patients were hospitalized for one day for duplex ultrasound evaluation and physical examination to monitor complications. All patients were encouraged to begin walking as soon as possible, and were also encouraged to use compression stockings post EVLA for at least 2-3 weeks or as long as possible. Additionally, a short course (5 days) of paracetamol 500 mg TID, ibuprofen 200 mg BID, and cetirizine 10 mg OD was prescribed to relieve symptoms, on top of their routine outpatient therapy.

### Postprocedural evaluation

All patients underwent outpatient follow-up evaluations, consisting of physical examination and duplex ultrasound assessment on day 7, at week 4, 8, and 12 after the procedure. Any potential complications were recorded., such as vein obliteration, thrombophlebitis, bleeding, hematoma, paresthesia, vein thrombosis, skin injury, and deep vein injury. Patients were advised to wear compression stockings for another 2 to 3 weeks.

### Statistical analysis

Continuous variables are presented as mean ± standard deviation (or median ± interquartile range), while categorical variables are expressed as frequency and percentage. All statistical analyses were performed using SPSS version 26 (IBM Corp), using 95% confidence intervals.

## Results

A total of 57 cases were eligible. The mean age of the patients was 61.61 years old (SD 9.576). Table 1 lists the number of patients stratified based on CEAP classification for CVI. Patients with CVI and a CEAP clinical substaging of C3 were the most patients included so far in our case series. Table 2 elaborates upon the average vessel diameter of the ablated vein in further detail, the LEED used, Laser Power/Energy and total treatment energy, procedure time, ablation length, and the amount of tumescent used. After a follow-up duration of 12 weeks, postoperative complication data were obtained. Among 57 cases that were eligible for this case series, there were 3 cases of postoperative leg pain (5.26%), 1 case of EHIT grade I-II (1,75%), 1 case of hyperpigmentation (1.75%), and 1 case of paresthesia during follow-up (1.75%). The average amount of time until complications were reported by the patient or found during routine examination was 13 days (SD 12.11). No cases were reported to have recanalized post EVLA with laser crossectomy, and follow-up results via duplex ultrasound had shown a completely obliterated great saphenous vein up from the saphenofemoral junction distally among all follow-up data. Table 3 elaborates further regarding postoperative outcomes during routine follow-up.

**Table 1.** Number of cases based on clinical CEAP classification.

	Frequency	Percent	Cumulative percent
**C2**	12	21,1	21,1
**C3**	40	70,2	91,2
**C4**	4	7,0	98,2
**C5**	1	1,8	100,0
Total	57	100,0	100,0

**Table 2.** Descriptive data regarding average vessel diameter of the ablated vein, LEED used, laser power/energy used, total treatment energy, procedure time, ablation length, amount of tumescent used.

	Mean	Standard Deviation (SD)
**Vessel diameter, SFJ (mm)**	7,395	1,8541
**Average vessel diameter, ATK (mm)**	5,39061	1,484488
**Average vessel diameter, BTK (mm)**	2,84696	0,668719
**Ablation length (cm)**	59,868	8,9465
**First Segment SFJ total laser energy (J)**	150 J	
**Total treatment energy, ATK+BTK (J)**	2021,225	474,8443
**Targeted Laser Power ATK**	6-7 W	**-**
**Targeted Laser Power BTK**	4-5 W	**-**
**Targeted LEED ATK**	50-70 J/cm	**-**
**Targeted LEED BTK**	20-30 J/cm	**-**
**Pullback speed**	0.14 s/cm = 1/7 s per cm	**-**

**Table 3.** Postoperative outcomes during routine follow-up after EVLA with laser crossectomy.

	Number of cases	Mean	SD	Percentage
**EHIT**	1	1,00	0,000	1.5%
**Thrombophlebitis**	0	0	0	0
**Hematoma**	0	0	0	0
**Hyperpigmentation**	1	1,00	0	1,754386
**Paresthesia**	1	1,00	0	1,754386
**Deep vein thrombosis**	0	0	0	0
**Thermal injury**	0	0	0	0
**Deep vein injury**	0	0	0	0
**Recanalization**	0	0	0	0
**Time till complication (days)**	4	13,00	12,111	7,017544

## Discussion

This case series highlights our single-center experience in conducting zone-zero laser crossectomy in EVLA patients, as well as post-operative outcomes during routine follow-up. Zone-zero laser crossectomy has become a standard protocol in our center of excellence. Previously, GSV recanalization had been an issue for conventional EVLA, due to a residual stump up to the SFJ, which can predispose to venous blood reflux, reopening the previously obliterated and failure to obliterate the pathway from the epigastric vein. With zone-zero laser crossectomy, we aimed to achieve total obliteration of the SFJ exactly in the SFJ, in an attempt to achieve complete obliteration and avoid stump formation in the SFJ.

After 3 months of follow-up, no patients were found to have recanalized GSV or Small Saphenous Vein (SSV) post EVLA with zone-zero laser crossectomy. One meta-analysis estimates the incidence rate of recurrence of varicose veins to be 6.82% amongst five studies, occurring on average one year after surgery. ^6^ Amongst our 3-month follow-up duration, our pooled rates for varicose vein recurrence or recanalization were 0%. When compared to flush-EVLA, another meta-analysis estimated that amongst 345 pooled patients, the pooled incidence rates were 9.25% for flush-EVLA and 19.29% for standard EVLA, respectively. ^7^ No cases of recanalization post EVLA were found during follow-up among all the samples included. The prevalence of EHIT post EVLA found in our case series was 1.5%. This range coincides with the estimates in prior literature that estimate the prevalence of EHIT in 1-18%. ^8^ Postoperative leg pain post EVLA is to be expected; however, the exact prevalence was poorly reported in prior studies. A recent study estimated that 13-17% experienced postoperative leg pain at any point after EVLA, and another study estimated that the prevalence of postoperative leg pain was reported to be 9-40%. ^9^ Neuropathy is also one of the more commonly reported outcomes, with other estimates ranging from 3-7% in prior studies. ^10^ Attempts to prevent nerve injury had been previously described, such as employing a two-step laser ablation protocol to reduce the risk of sural nerve injury after GSV ablation, as described by Utoh et al, however the concerns regarding potential nerve injury after EVLA still remain. ^11^ Currently, the prevalence of neuropathy post EVLA + zone-zero crossectomy in our case series lies at 1 case (1.57%), which even though it is lower than global estimates, however we also concur that the lack of total cases in our case series remains a major confounder, and that further follow-up data is required to derive more reliable estimates in the future.

This case series has several major limitations. Firstly, zone-zero crossectomy is a novel technique. Prior studies such as flush-EVLA, are described as ablating the saphenofemoral junction with target margins of 0-pulse distance (PD) up to −2 mm and +1 mm. Our experience with this new zone-zero technique is to aim the laser crossectomy right at the 0-PD zone of the saphenofemoral stump. This technique had recently become routine in our center only since the start of January 2025. We acknowledge that the lack of larger sample sizes could confound our findings in this case series. Second, the absence of longer follow-up data post-EVLA beyond 3 months is another limitation in our case series. We intend to conduct a follow-up study with a larger sample size and extended follow-up periods to address these gaps in the future.

## Consent

Before the procedure was conducted, an informed consent was given and signed by the patient or legal caretaker as per hospital protocols.

## Data Availability

1. Supplementary data used in this study is available in Figshare, under the title “Laser Crossectomy Supplementary Data for F1000”, with the working DOI: 10.6084/m9.figshare.30029992 ( https://figshare.com/articles/dataset/Laser_Crossectomy_Supplementary_Data_for_F1000/30029992), this content will be licensed as CC BY 4.0. ^13^
2.
Figure data is available Figshare, under the title “ Figure 1 - Bar Graph of Cases of CVI Based on CEAP Classification” with the working DOI: 10.6084/m9.figshare.30193801 ( https://figshare.com/articles/figure/Figure_1_-_Bar_Graph_of_Cases_of_CVI_Based_on_CEAP_Classification/30193801), this content will be licensed as CC BY 4.0. ^12^
3. The CARE checklist is available in Figshare, under the title “CARE Checklist for Laser Crossectomy” with the working DOI: 10.6084/m9.figshare.30193774, this content with be licensed as CC BY 4.0. ^14^ Available in: 10.6084/m9.figshare.30193801, https://figshare.com/articles/figure/Figure_1_-_Bar_Graph_of_Cases_of_CVI_Based_on_CEAP_Classification/30193801. CVI: Chronic Venous Insufficiency; CEAP: Clinical, Etiology, Anatomical, Pathology classification; C2: Varicose veins; C3: edema; C4: skin discoloration; C5: Ulcer. Figures must be uploaded to the submission as separate TIFF or JPEG files at >300dpi. Ensure any abbreviations/acronyms in the tables are explained in the legend.
