# Supplementary material for: Successful single-center experiences of laser crossectomy in zone zero in Endovenous Laser Ablation (EVLA) for chronic venous insufficiency - a case series
Source: F1000Res. 2026 Jan 21;15:96. [Version 1] doi: 10.12688/f1000research.170645.1 (PMC12982993; doi:10.12688/f1000research.170645.1)
Supplement: Peer review history

# Reviewer report: Reviewer response for version 1

**DOI:** 10.5256/f1000research.188133.r457944

**Published:** 2026-03-12

**Authors:** Yopie Afriandi Habibie

**Affiliations:** 1 Thoracic Cardiac and Vascular Surgery, University of Syiah Kual, Banda Aceh, Aceh, Indonesia

This study introduces zone-zero laser crossectomy, a new technique aimed at addressing residual stumps and recurrence in conventional endovenous laser ablation (EVLA). It reports a 100% occlusion rate and low complication rates at the 12-week follow-up. The authors acknowledge limitations, including a small sample size and short follow-up period, which enhance the credibility of their findings.

 Area of Improvement :

 1. **Longer Follow-Up**: The study only includes a 12-week follow-up period, which is insufficient for assessing the technique's long-term durability. I suggest revising the title to reflect early outcomes.

 2. **Comparison with Other Techniques:** Although the study mentions conventional EVLA and flush-EVLA, it does not present any direct comparative data. Including a control group or comparing the outcomes with other techniques would strengthen the study's impact. Is there a specific reason for the lack of comparison?

 3. **Mechanisms of Recurrence**: The discussion could benefit from a more detailed explanation of recurrence mechanisms, such as neovascularization and residual reflux, to strengthen the rationale for the technique.

 4. Please include a few images in the post-operative USG to show improvement after zero-zero crossectomy. This will be more beneficial for readers.

Are enough details provided of any physical examination and diagnostic tests, treatment given and outcomes?

Yes

Is the background of the cases’ history and progression described in sufficient detail?

Yes

Is sufficient discussion included of the importance of the findings and their relevance to future understanding of disease processes, diagnosis or treatment?

Partly

Is the conclusion balanced and justified on the basis of the findings?

Yes

Reviewer Expertise:

Cardiovascular surgery, Thoracic Surgery, Vascular and Endovascular, Emergency Medicine, Critical Care

I confirm that I have read this submission and believe that I have an appropriate level of expertise to confirm that it is of an acceptable scientific standard.

# Reviewer report: Reviewer response for version 1

**DOI:** 10.5256/f1000research.188133.r457948

**Published:** 2026-02-28

**Authors:** Johanes Nugroho

**Affiliations:** 1 Airlangga University, Surabaya, East Java, Indonesia

The background clearly explains the clinical burden of chronic venous insufficiency, the rationale for conventional EVLA margins, and the theoretical advantages of zone-zero laser crossectomy. The manuscript appropriately highlights stump-related recurrence and positions this technique as a minimally invasive evolution of high ligation. A brief expansion on recurrence mechanisms (neovascularization vs residual reflux) could further strengthen the rationale.

 The work is clearly structured and well presented. The procedural description is detailed and reproducible, and relevant literature—including contemporary guideline frameworks—is appropriately cited. Minor refinements in decimal formatting and Table 2 presentation would improve clarity.

 As a descriptive case series, the statistical analysis is appropriate. Results are interpreted cautiously, and limitations related to short follow-up are acknowledged. Inclusion of confidence intervals would enhance completeness but is not essential.

 The procedural protocol is described in excellent technical detail, allowing practical reproducibility. Clarification of duplex criteria for defining complete obliteration and inclusion of full descriptive ranges in Table 2 would further enhance transparency.

 The conclusions are adequately supported by the reported early outcomes. The 100% early occlusion rate and low complication profile support the authors’ statements within the 12-week follow-up context.

 Overall, this manuscript provides valuable preliminary data and practical insight into zone-zero EVLA. It represents a meaningful technical contribution to evolving endovenous practice and merits publication following minor revisions.

Are enough details provided of any physical examination and diagnostic tests, treatment given and outcomes?

Yes

Is the background of the cases’ history and progression described in sufficient detail?

Yes

Is sufficient discussion included of the importance of the findings and their relevance to future understanding of disease processes, diagnosis or treatment?

Yes

Is the conclusion balanced and justified on the basis of the findings?

Yes

Reviewer Expertise:

Cardiovasvular

I confirm that I have read this submission and believe that I have an appropriate level of expertise to confirm that it is of an acceptable scientific standard.
